# Validity and Reliability of the Japanese Version of the painDETECT Questionnaire: A Multicenter Observational Study

**DOI:** 10.1371/journal.pone.0068013

**Published:** 2013-09-30

**Authors:** Yoshitaka Matsubayashi, Katsushi Takeshita, Masahiko Sumitani, Yasushi Oshima, Juichi Tonosu, So Kato, Junichi Ohya, Takeshi Oichi, Naoki Okamoto, Sakae Tanaka

**Affiliations:** 1 Department of Orthopaedic Surgery, Faculty of Medicine, the University of Tokyo, Tokyo, Japan; 2 Department of Anesthesiology and Pain Relief Center, the University of Tokyo, Tokyo, Japan; Tokyo Metropolitan Institute of Medical Science, Japan

## Abstract

**Objectives:**

The aim of this study was to evaluate the validity and reliability of the Japanese version of the painDETECT questionnaire (PDQ-J).

**Materials and Methods:**

The translation of the original PDQ into Japanese was achieved according to the published guidelines. Subsequently, a multicenter observational study was performed to evaluate the validity and reliability of PDQ-J, including 113 Japanese patients suffering from pain.

**Results:**

Factor analysis revealed that the main component of PDQ-J comprises two determinative factors, which account for 62% of the variance observed. Moreover, PDQ-J revealed statistically significant correlation with the intensity of pain (Numerical Rating Scale), Physical Component Score, and Mental Component Score of the Medical Outcomes Study 36-Item Short-Form Health Survey (SF-36). The Cronbach alpha for the total score was 0.78 and for the main component was 0.80. In the analysis of test–retest method, the intraclass correlation coefficient between the two scores was 0.94.

**Conclusions:**

We demonstrated the validity and reliability of PDQ-J. We encourage researchers and clinicians to use this tool for the assessment of patients who suffer suspected neuropathic pain.

## Introduction

Neuropathic pain is defined as “pain caused by a lesion or disease of the somatosensory system” [1], and its prevalence reaches about 7%–8% in the European population [2,3]. A variety of diseases such as diabetic polyneuropathy, postherpetic neuralgia, spinal cord injury, and peripheral nerve compression cause neuropathic pain, and they generally follow a chronic course. Chronic pain in patients results in anxiety, depression, and interference with sleep, normal work, and social activities [4,5]. It has a strong negative impact on the quality of life [6] and has been estimated to result in an expense of more than $100 billion per year in direct medical costs and about $100 billion as indirect costs from absenteeism and decreased productivity at work in the United States [7]. Among chronic pain conditions, neuropathic pain impairs the quality of life profoundly, and patients with neuropathic pain are likely to generate more expenses compared with those with other pain conditions [8]. Although early and intense care of neuropathic pain is important, diagnosing neuropathic pain is a challenge because lesions of the somatosensory nervous system are not readily detectable. Unlike non-neuropathic pain conditions, neuropathic pain usually reveals characteristic symptoms such as “burning sensation,” “prickling sensation,” and/or a sensation of “electric shock [9].” On the basis of such characteristic descriptions, screening tools have been developed to identify the components of neuropathic pain from a patient’s presentation of symptoms.

**Figure 1:**
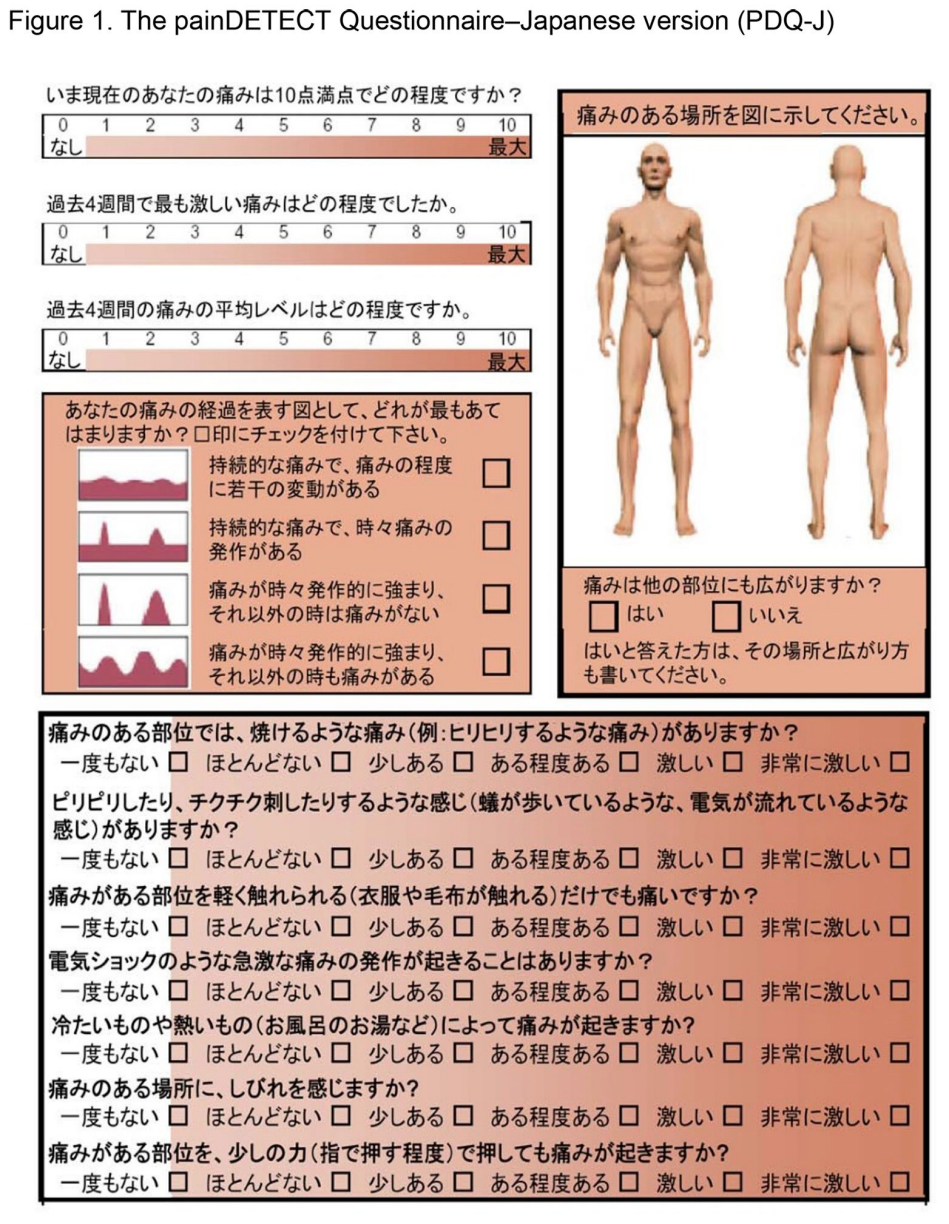
The painDETECT Questionnaire-Japanese version (PDQ-J).

The painDETECT questionnaire (PDQ) is one of the screening tools of neuropathic pain, which was published by Freynhagen et al. from Germany [10,11]. They established the usefulness and validity of this brief, self-administered questionnaire in identifying neuropathic components of pain in patients with chronic lower back pain. PDQ has already been translated into Spanish [12], Dutch [13], and Japanese [14]. The Japanese version of PDQ (PDQ-J) was translated and reported by one of the authors of the present study in 2007 (Figure 1). Translation and cross-cultural adaptation of PDQ-J was performed in accordance as per the established guidelines [15,16]. First, for forward translation, a professional native Japanese translator and a bilingual Japanese physician independently translated the original PDQ into Japanese. Second, an expert committee, including specialists in pain management, orthopedics, and methodology, conducted synthesis of the translation. Third, two native English translators, who were uninformed about the nature of the study, completed back translations of the translated PDQ; thereafter, back-translations were sent to the expert committee to detect any existing cultural bias, and the final version of PDQ-J was completed. Nevertheless, the validity of PDQ-J has not been confirmed yet; therefore, this study aimed to assess the validity and reliability of PDQ-J.

## Materials and Methods

The study protocol was approved by the institutional review board of the Clinical Research Support Center of the University of Tokyo Hospital. Participants provided their written informed consent to participate in this study.

We conducted a multicenter observational study, and patients from two adult populations were enrolled. All the enrolled patients suffered from pain with an intensity of 3 or more out of 10 on an 11-point numerical rating scale (NRS). The first study group included patients with neuropathic pain (NeP group) diagnosed by a pain specialist in the pain center as per the guidelines established by the International Association for the Study of Pain (IASP) [17]. In the pain center, only neuropathic patients with stable disease condition and tolerable pain were selected; in addition, they were selected if it could be estimated that there would be little change in their pain during the study period. The second study group comprised patients suffering from acute nociceptive pain (NocP group) induced by trauma or orthopedic patients with a degenerative condition of the extremities. Moreover, patients with cultural or language barriers or with poor mental health status that prevented them from understanding or responding to proposed questions were excluded from this study. Informed consent was provided by selected patients from both the groups. In the first survey, all patients were asked to complete a set of questionnaires including PDQ-J, a three-type numeric rating scale (NRS) of pain (i.e., pain during the survey, a four-week average, and maximum pain experienced), and the Medical Outcomes Study 36-Item Short-Form Health Survey (SF-36). The patients answered questions regarding their demographic data (e.g., age, sex, height, weight, occupation, smoking history, and education). Thereafter, the physicians reported the original diagnoses, comorbidities, and treatment options. The second survey questionnaire was administered only to the neuropathic patients 2–5 weeks after the first visit, and it included the same set of three questionnaires with one additional question regarding whether there was an increase, decrease, or no change in pain since the administration of the first survey.

PDQ comprises a main component along with two additional components. In the main component, termed as “gradation of pain,” the patient is asked to identify the presence of seven pathological pain sensations: burning, tingling, or prickling sensations, tactile and thermal allodynia, electric shock-like sensations, numbness, and pressure-evoked pain sensation. The patient grades the presence of each type of pain as follows: 0 = never; 1 = hardly noticed; 2 = slightly; 3 = moderately; 4 = strongly; 5 = very strongly. This main component of PDQ yields scores from 0 to 35 points. A second component of PDQ, termed as “pain course pattern,” is a multiple-choice questionnaire accompanied by four pain charts; the patient is asked to quantify the pattern of experienced pain as follows: persistent pain with slight fluctuations (0 points); persistent pain with pain attacks (−1 point); pain attacks without pain between them (1 point); pain attacks with pain between them (1 point). The third component of PDQ, termed “radiating pain,” asks patients regarding radiation of pain to other regions of the body (2 points). A total score is calculated by adding the scores from the three components; a high score indicates that the pain is possibly neuropathic in nature.

The intensity of pain was assessed by a three-type NRS where the patient is asked to grade the actual pain level experienced, the maximum pain level experienced in the last four weeks, and the average pain level experienced in last four weeks on a scale of 0-10 (0 = no pain, 10 = worst pain imaginable).

The SF-36 consists of eight subscales, namely physical functioning, physical role functioning, bodily pain, general health perceptions, vitality, social role functioning, emotional role functioning, and mental health [18,19]. Each subscale is transformed to a score ranging from 0 to 100, with lower scores indicating poor health-related quality of life. For analysis, we used two summed scores: the Physical Component Score (PCS) and the Mental Component Score (MCS). Each score has the same mean and standard deviation (50 and 10, respectively) in a normal population.

### Feasibility

We analyzed the number of unanswered questions to evaluate the feasibility of PDQ-J.

### Validity

To establish construct validity, we performed an exploratory factor analysis with principal components extraction. The Kaiser criterion (eigenvalues > 1.0) and Scree plot were used to determine the number of factors. As for criterion-related validity, we calculated the Pearson correlation coefficient between PDQ-J, NRS, PCS (SF-36), and MCS (SF-36). Following are the generally accepted rankings for coefficients: 1.0–0.81 (excellent); 0.80–0.61 (very good); 0.60–0.41 (good); 0.40–0.21 (fair); and 0.20–0 (poor) [20].

### Reliability

Internal consistency was measured with Cronbach’s alpha. Alpha coefficients of a magnitude of ≥0.70 were considered useful as evidence of adequate scale reliability at the level of group comparisons [21]. Repeatability was assessed by a test–retest method. Retest was administered to neuropathic patients more than two weeks after first survey. Intraclass correlation coefficients (ICCs) between test and retest scores were calculated from the data from patients who responded with no change of symptoms between the two surveys; moreover, those with coefficients >0.80 were considered as having excellent reliability [22].

All statistical analyses were performed using the Statistical Package for the Social Sciences (SPSS version 21.0) software.

## Results

A total of 122 Japanese patients were recruited in this study. However, nine patients were excluded because of incomplete responses to the proposed questions; most (six of nine) of the blank responses were to the question regarding the radiation of pain. Following exclusions, a total of 113 patients were further evaluated: 60 patients were diagnosed as having neuropathic pain, and 53 were categorized as having nociceptive pain. The demographic characteristics of these patients are presented in Tables 1, 2, and 3; in addition, it lists specific etiologies of pain in patients in the NeP group [brachial plexus injury (12 patients); radiculopathy (12 patients); herpes zoster (11 patients); spinal cord injury (10 patients); diabetic or alcoholic polyneuropathies (7 patients); phantom pain (5 patients); complex regional pain syndrome (CRPS; 2 patients); carpal tunnel syndrome (1 patient); and thalamic pain (1 patient)] and the NocP group [trauma in 47 patients (89%), and degenerative diseases in 6 patients (11%)] (Table 4). Specific etiologies included fractures (32 patients), contusion/sprains (10 patients), osteoarthritis (4 patients), muscle pain (3 patients), dislocations (2 patients), tenosynovitis (1 patient), and rotator cuff injury (1 patient).

**Table 1:** Demographic data of study patients.

	**NeP (n=60) (SD)**	**NocP (n=53) (SD)**	**P value (t-test)**
**Age (mean)**	59 (15)	57 (18)	n.s.
**Male/Female**	40/20	30/23	n.s.
**Height (mean)**	164 (10)	164 (11)	n.s.
**Weight (mean)**	64 (17)	62 (13)	n.s.
**Duration (months)**	63 (71)	2.3 (7.9)	<0.001

NeP: Neuropathic Pain, NocP: Nociceptive Pain

**Table 2:** Demographic data of study patients (education).

**Education**	**NeP (n)**	**NocP (n)**
**Middle school**	8	11
**High school**	24	14
**Junior college**	6	13
**University**	16	15
**Graduate university**	4	0

NeP: Neuropathic Pain, NocP: Nociceptive Pain

**Table 3:** Demographic data of study patients (occupation).

**Occupation**	**NeP (n)**	**NocP (n)**
**Employee**	15	18
**Retired**	19	12
**Self-employed**	11	9
**Housewife**	8	8
**Part-time job**	3	6
**Student**	1	0
**Employer**	1	0

NeP: Neuropathic Pain, NocP: Nociceptive Pain

**Table 4:** Etiology of study patients.

**Neuropathic Pain**	**Nociceptive Pain**
Brachial Plexus Injury 12, Radiculopathy 12, Herpes zoster 11, Spinal cord injury 10, Neuropathy 7, Phantom pain 5, complex regional pain syndrome 2, Carpal Tunnel Syndrome 1, Thalamic pain 1	Fracture 32, Contusion/sprain 10, Osteoarthritis 4, Muscle pain 3, Dislocation 2, Tenosynovitis 1, Rotator cuff injury 1

Tables 5 and 6 presents the summary of patient responses of PDQ-J, and Table 7 presents the scores for each questionnaire in NeP and NocP group, respectively. On the pain intensity scale, patients from the NeP group experienced pain that was significantly more severe compared with that in the NocP group. The PDQ-J and SF-36 scores revealed similar trends: patients in the NeP group revealed lower physical and mental functioning compared with that in patients in the NocP group.

**Table 5:** PainDETECT Questionnaire-Japanese version (PDQ-J): summary of patient responses (Q1-7).

**n (NeP/NocP)**	**No**	**Hardly noticed**	**Slightly**	**Moderately**	**Strongly**	**Very strongly**
**Q1. Burning**	14 (4/10)	25 (11/14)	30 (9/21)	21 (17/4)	18 (14/4)	5 (5/0)
**Q2. Tingling**	10 (0/10)	12 (6/6)	39 (13/26)	25 (17/8)	19 (18/1)	8 (6/2)
**Q3. Pain by touch**	17 (8/9)	30 (15/15)	24 (10/14)	21 (12/9)	15 (12/3)	6 (6/2)
**Q4. Electric shock-like pain**	28 (9/19)	34 (16/18)	18 (11/7)	18 (12/6)	9 (8/1)	6 (4/2)
**Q5. Pain on cold/hot stimulation**	33 (8/25)	40 (23/17)	19 (12/7)	15 (12/3)	2 (2/0)	4 (3/1)
**Q6. Numbness**	15 (1/14)	23 (5/18)	19 (7/12)	19 (13/6)	18 (17/1)	19 (17/2)
**Q7. Pain by pressure**	5 (3/2)	28 (18/10)	22 (8/14)	26 (14/12)	19 (10/9)	13 (7/6)

NeP: Neuropathic Pain, NocP: Nociceptive Pain

**Table 6:** PainDETECT Questionnaire-Japanese version (PDQ-J): summary of patient responses (Q8-9).

**Q8. Pain course pattern**	**n (NeP/NocP)**
Persistent pain with slight fluctuations	46 (22/24)
Persistent pain with pain attacks	21 (12/9)
Pain attacks without pain between them	18 (3/15)
Pain attacks with between them	28 (23/5)
**Q9. Radiating pain**	**n (NeP/NocP)**
Yes	74 (35/39)
No	34 (23/11)

NeP: Neuropathic Pain, NocP: Nociceptive Pain

**Table 7:** Scores of Pain Intensity, Neuropathic pain, and health-related outcomes.

**Outcomes**	**NeP (SD)**	**NocP (SD)**	**P-value (t-test)**
**Pain Intensity-NRS (present)**	6.5 (2.3)	4.3 (2.9)	< 0.001
**Pain Intensity-NRS (average)**	6.7 (2.0)	4.2 (2.3)	< 0.001
**Pain Intensity-NRS (maximum)**	8.3 (1.6)	6.6 (3.2)	< 0.001
**painDETECT**	18.6 (6.3)	11.8 (6.3)	< 0.001
**PCS (SF-36)**	26.6 (16.9)	34.3 (20.8)	< 0.05
**MCS (SF-36)**	41.6 (11.7)	52.7 (10.2)	< 0.001

NeP: Neuropathic Pain, NocP: Nociceptive Pain, NRS: Numerical Rating Scale, SF-36: Medical Outcomes Study 36-Item Short-Form Health Survey, PCS: Physical Component score, MCS: Mental Component Score

### Validity

The factor analysis by Promax rotation using the Kaiser criterion (eigenvalues ≥1.0) and a Scree plot revealed that the main component of PDQ-J consists of two determinative factors, and it explained 62% of the variance. One of these determinative factors can be termed as “spontaneous pain,” and the other as “evoked pain.” For criterion-related validity, the total score of PDQ-J revealed statistically significant correlations with pain intensity, PCS (SF-36), and MCS (SF-36; Table 8).

**Table 8:** Pearson correlation coefficient with PDQ-J.

	**PDQ-J**	**P Value**
**Pain-Intensity (NRS)**	0.44	< 0.01
**PCS (SF-36)**	−0.27	< 0.01
**MCS (SF-36)**	−0.34	< 0.01

NeP: Neuropathic Pain, NocP: Nociceptive Pain, NRS: Numerical Rating Scale, SF-36: Medical Outcomes Study 36-Item Short-Form Health Survey, PCS: Physical Component score, MCS: Mental Component Score

### Reliability

**Figure 2:**
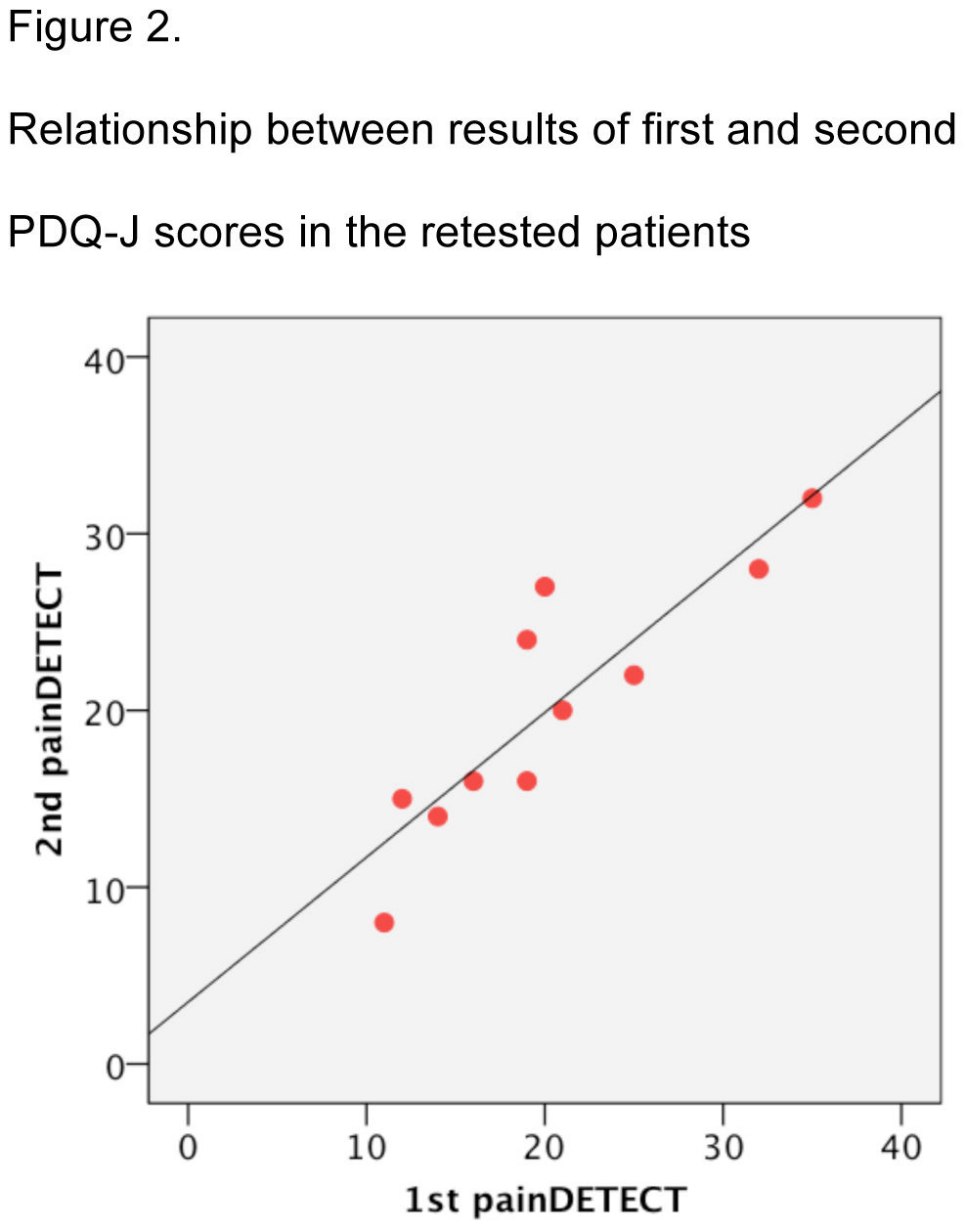
Relationship between results of first and second PDQ-J scores in the retested patients.

The Cronbach alpha for the total score of PDQ-J was 0.78 and that of the main component of PDQ (i.e., “gradation of pain”) was 0.80, which was comparable to 0.83 and 0.86 reported in the original and Spanish versions, respectively [11,12]. The score for each of the nine questions in PDQ-J revealed a statistically significant correlation with the total score of PDQ-J. We could recruit 16 patients with neuropathic pain for a test–retest study; of these, 11 patients reported no change in their symptoms, and the data for each these patients were evaluated. The average period between the two surveys was 23.1 days [standard deviation (SD): 8.3]. The mean score of the first and second survey was 20.4 (SD: 7.7) and 20.2 (SD: 7.2), respectively. Furthermore, ICC between the two scores was 0.94 despite the relatively long interval between the two surveys (Figure 2).

## Discussion

This study demonstrated that PDQ-J has good validity and reliability. In addition, the results obtained in this study were comparable with those obtained in previous studies [11,12]. With regard to construct validity, the factor analysis revealed that the seven Likert items of PDQ-J consist of two determinative factors, which could be designated as “spontaneous pain” and “evoked pain.” These factors are consistent with clinical characteristics of neuropathic pain. Further, with regard to criterion-related validity, the correlation between PDQ-J and NRS, MCS (SF-36), and PCS (SF-36) was moderate, indicating that PDQ-J can reflect pain intensity as well as impairments of mental status and physical status of individual patients. There is evidence from a previous study to support this, in which patients with more intense pain revealed higher total scores on PDQ [23]. Therefore, PDQ-J might be used as a score of pain severity, although another study should be conducted to validate this. In this study, we demonstrated fair to good criterion-related validity, excellent internal consistency, and high reliability with statistical significance, although the number of patients was limited, particularly in the analysis of repeatability. As this study evaluated two distinct types of pain, neuropathic and acute nociceptive pain in the extremities, the methods and results obtained in this study might be useful in a wide patient population suffering from various types of pain.

Because the prevalence of patients with neuropathic pain is limited in general population [24], neuropathic pain has not been still recognized well in clinical settings all over the world and also in Japan. However, the indications are that this type of pain is more severe than non-neuropathic pain and results in profound impairment of both physical and mental quality of life. In addition, neuropathic pain is usually resistant to treatment with conventional analgesic medications such as acetaminophen and nonsteroidal anti-inflammatory drugs (NSAIDs), and yet, it is not uncommon that such ineffective measures are prescribed for patients suffering from neuropathic pain. On the other hand, it is well known that specific medications such as tricyclic antidepressants and pregabalin/gabapentine provide effective analgesia in patients with neuropathic pain. Therefore, the detection of a neuropathic pain component from a patient’s total pain presentation is important in selecting the appropriate medication for appropriate pain management; this is particularly true for general physicians without expertise in pain management. Screening tools for identifying neuropathic pain, such as PDQ, have revealed that a neuropathic pain component is underdiagnosed in a profound number of patients with pain, thereby suggesting that patients with neuropathic pain are not administered analgesics that are most effective in treating this type of pain. Similar circumstances are probably present in Japan as well. The results of the present study along with confirmed reliability and validity of PDQ-J provide the rationale to encourage extension of its use by general physicians in Japan to promote appropriate pain management in patients suffering with conditions involving chronic pain.

## Conclusion

This study confirms that PDQ-J has good reliability and validity as a pain assessment tool. Thus, we encourage researchers and clinicians to use PDQ-J for the assessment of patients suffering from pain that is suspected to be neuropathic in origin.
